# Contrasting Mind-Wandering, (Dark) Flow, and Affect During Multiline and Single-Line Slot Machine Play

**DOI:** 10.1007/s10899-021-10027-0

**Published:** 2021-05-06

**Authors:** Tyler B. Kruger, Mike J. Dixon, Candice Graydon, Chanel J. Larche, Madison Stange, Stephen D. Smith, Daniel Smilek

**Affiliations:** 1 Department of Psychology, University of Waterloo, 200 University Avenue West, Waterloo, ON N2L 3G1 Canada; 2 Gambling Research Lab, University of Waterloo, 200 University Avenue West, Waterloo, ON N2L 3G1 Canada; 3 Department of Psychology, University of Winnipeg, 515 Portage Avenue, Winnipeg, MB R3B 2E9 Canada

**Keywords:** Mindfulness, Depression, Dark flow, Mind-wandering, Gambling

## Abstract

Slot machines are a very popular form of gambling in which a small proportion of gamblers experience gambling-related problems. These players refer to a trance-like state that researchers have labelled ‘dark flow’—a pleasurable, but maladaptive state where players become completely occupied by the game. We assessed 110 gamblers for mindfulness (using the Mindful Attention Awareness Scale), gambling problems (using the Problem Gambling Severity Index), depressive symptoms (using the Depression, Anxiety, and Stress Scale), and boredom proneness (using the Boredom Proneness Scale). Participants played both a multiline and single-line slot machine simulator and were occasionally interrupted with thought probes to assess whether they were thinking about the game or something else. After playing each game, we retrospectively assessed dark flow and affect during play. Our key results were that the number of “on-game” reports during the multiline game were significantly higher than the single-line game, and that we found significantly greater flow during the multiline game than the single-line game. We also found significantly lower negative affect during the multiline game than the single-line game. Using hierarchical multiple regression, we found that dark flow accounted for unique variance when predicting problem gambling severity (over and above depression, mindfulness, and boredom proneness). These assessments help bolster our previous assertions about escape gambling—if some players are prone to having their mind-wander to negative places, the frequent but unpredictable reinforcement of multiline slot machines may help rein in the wandering mind and prevent minds from unintentionally wandering to negative thoughts.

## Introduction

In our home jurisdiction of Ontario, Canada, the Ontario Lottery and Gaming Corporation (OLG) is a government body responsible for conducting and managing casinos, gaming facilities, and lotteries. According to the 2018–2019 annual report, the OLG generated approximately $8.3 billion in total revenue with approximately half (46.5%) coming from land-based gaming where slot machines are housed (Ontario Lottery and Gaming Corporation, 2019). In a report conducted for the Ontario Problem Gambling Research Centre and the Ontario Ministry of Health and Long-Term Care, Willams and Volberg (2013) estimated that problem gamblers account for approximately one quarter (24.1%) of the revenue generated from government-sponsored gambling. They also note that the proportion of revenue generated from problem gamblers playing electronic gambling machines (e.g., slot machines) is even higher (Williams & Volberg, 2013). Researchers are unified in the view that slot machines can create problems for some individuals (Dowling et al., 2019; Pfund et al., 2020)—for example, in 2017, the Ontario Problem Gambling Helpline received more calls from gamblers regarding slot machines than any other type of gambling (Graydon et al., 2017). Thus, it is essential to understand why slot machines have the propensity to create such problems and which players are most likely to be negatively impacted by slots play.

Slot machines have a unique appeal compared to other modes of gambling. For example, unlike lotteries, where players may have to wait days before knowing whether they won or lost, in slots play, feedback is immediate. When a player spins and wins, the machine celebrates the win by providing the player with feedback and reinforcement in the form of jingles and animations—with the length of the feedback proportional to the win size. Conversely, when the player spins and loses their entire wager, the machine goes into a state of quiet and the player isn’t provided with sounds or animations. On single-line slot machines these are the only outcomes that a player can receive. In single-line games, slots play is characterized by long chains of losses where the machine remains quiet. These prolonged losing streaks are occasionally interrupted by wins and their accompanying sights and sounds (Dixon et al., 2014a, 2014b). However, in most modern machines, players can bet on multiple lines per spin. The lines can be either horizontal, vertical, diagonal, or form a zig-zag pattern. Given the complexity of these combinations, it can be very difficult for players to tell if they won or lost based on the machine’s end-of-spin symbol arrangement alone. Thus, many players attend to the high-fidelity attention-grabbing sights and sounds to tell if they won or lost money on that spin (Griffiths & Parke, 2005; Haas & Edworthy, 1996). In addition to the regular losses and wins found on single-line slot machines, on multiline slots there is a third and arguably problematic outcome called a ‘loss disguised as a win’ or LDW (Dixon et al., 2010). For example, if a player bets $1.00 spread across a number of lines, but only wins back $0.20, the machine still provides the player with reinforcing sights and sounds despite the player incurring a net loss of $0.80—the machine essentially celebrates the fact that the player lost money on that spin. LDWs are more frequent than actual wins (Dixon et al., 2010) and on some slot machines, players will experience reinforcing stimuli almost every other spin (Dixon et al., 2014a, 2014b). Thus, relying on the machine’s feedback to tell if you won or lost can be problematic since the majority of novice players believe that LDWs are true wins (Jenson et al., 2013). Even frequent slot players tended to overestimate the number of times they actually won credits during a multiline slots session—likely an effect of conflating true wins with LDWs in memory (Dixon et al., 2014a, 2014b).

Dixon et al. (2014a), (2014b) investigated whether players preferred multiline slot machines over single-line machines and how players interacted with the different machines. The researchers had players bet either 1-cent on a single-line (1-cent per spin) or bet 1-cent on each of 20-lines (20-cents per spin). Despite losing more on the 20-line game, the majority of players still preferred the multiline game over the single-line game. Dixon and colleagues replicated this preference for multiline games in another study in which they equated the total bet per spin for the single and multiline games (Dixon et al., 2017). In one session they had slots players bet 1-cent on each of 20-lines on a “penny” machine for a total bet size of 20-cents. In another session, players bet 4 credits on a “nickel” machine where each credit was 5-cents for a total bet size of 20-cents. Once again, they found that players preferred the multiline game over the single-line game. Playing the maximum number of lines with a minimum bet per line (the so-called “maximin strategy”) appears to be a strategy favoured by frequent players (Williamson & Walker, 2001; Walker, 2004; Livingstone et al., 2008; Templeton et al., 2015).

Some slot machine players describe entering a trance-like state while playing the slot machine, a feeling which they call the “slot machine zone” (Schüll, 2005; Murch et al., 2017). Gamblers report a strong desire to be alone in order to enter this “zone” and once in this state, problem gamblers become so absorbed with the machine that they experience an extreme narrowing of attention and feelings of positive affect (Diskin & Hodgins, 1999, 2001; Dixon et al., 2019a, 2019b; Murch et al., 2017). This extreme narrowing of attention and trance-like state that gamblers describe is somewhat reminiscent of flow states referred to in positive psychology: total engagement with the current environment to the point where attending to task-relevant stimuli is effortless (Csikszentmihalyi & Csikszentmihalyi, 1992; Marty-Dugas & Smilek, 2018). Although flow is typically viewed as something favorable, Dixon and colleagues (2017/2019) refer to the slot machine zone as a state of “dark flow” because of the potentially negative consequences this state engenders for the player (e.g., spending more time or money than initially planned at the slot machine). For a comprehensive review of this highly immersive state and its relation to dissociative experiences and problem gambling see Schluter and Hodgins (2019).

Dark flow may also help alleviate symptomology attributable to mood disorders such as anxiety and/or depression. In fact, researchers have shown a reliable association between mood disorders and problem gambling status (Dixon et al., 2017, 2019a, 2019b). Underlying this relation, it appears that a subset of problem gamblers may use gambling as a way to self-medicate their depressive symptomology (Abbott & Volberg, 1996; Blaszczynski et al., 1990; Getty et al., 2000; Blaszczynski & Nower, 2002; Griffiths & Auer, 2013, Dixon et al., 2017, 2019a, 2019b). A study conducted by Dixon et al. (2017) investigated the relationships between dark flow, depression, and problem gambling status during multiline slot-machine play. Dark flow was characterized by distortion of time and engagement with the slot machine. They found that problem gambling severity, measured by the Problem Gambling Severity Index (PGSI), correlated with flow scores from the Game Experiences Questionnaire (GEQ; *r*(134) = .57). Flow scores also were significantly correlated with depression scores measured by the depression subscale of the 21-item Depression, Anxiety, and Stress Scale, (*r*(134) = .51). In a more recent study, they replicated these relations between problem gambling status and flow, *r*(127) = .25, and depression and flow, *r*(127) = .46 (Dixon et al., 2019a, 2019b). Taken together, individual differences in the propensity to become immersed during slot machine play may contribute to the development of pathological gambling. Depressed players may find themselves in the highly pleasurable “slot machine zone”—a flow state which provides an escape and relief from ruminating about their depressed lives.

Mind-wandering also impacts mood. Mind-wandering occurs when attention is shifted away from the current task in the external environment towards unrelated self-generated internal thoughts (Bertossi et al., 2017; Seli et al., 2018; Smallwood & Schooler, 2006). Mind-wandering is estimated to occupy almost half of all waking activity and is associated with negative affect, whereas an occupied mind generates positive affect (Killingsworth & Gilbert, 2010). Mindfulness (the antithesis of mind-wandering) is present-focused and refers to moment-to-moment mental awareness of one’s emotions, bodily sensations, and mental states (Bishop et al., 2004; Wheeler et al., 2017). An important characteristic of mindfulness involves directing and focusing attention. Previous research has shown that problem gamblers score lower on measures of mindfulness than their non-problem counterparts (de Lisle et al., 2011; Dixon et al., 2019a, 2019b; Reid et al., 2014).

Dixon et al. (2019a, 2019b) replicated the finding that mindfulness in everyday life (assessed using the 15-item Mindfulness Attention Awareness Scale; MAAS) was negatively correlated with problem gambling severity, *r*(127) = − .49 (i.e., the more mindfulness problems the greater the problem gambling severity). The authors suggest that gamblers with mindfulness problems in everyday life may find their attention locked in by the slot machine, which induces dark flow and ultimately facilitates positive affect. Dixon et al. (2019a, 2019b) interrupted slots play to assess mindfulness by asking if the participants were either focused on the game (i.e., they were on-task) or if they were thinking about something else (i.e., mind-wandering) just before the thought probe appeared. They found that the strong correlation between problem gambling status and mindfulness problems in everyday life, was eliminated in the slot machine context. When players were probed during slots play, there was no correlation between problem gambling status and their propensity to mind wander *while playing slots—*presumably because the intermittently celebratory feedback from the machine tended to rein in their attention. The reining in of attention during multiline slots play also might have the potential to explain why frequent players prefer multiline games over single-line games—a hypothesis we will test in this study. In single-line games where there are long chains of losses, players may find their minds wandering. If wandering minds are unhappy minds, as suggested by Killingsworth and Gilbert (2010), such mind-wandering may induce negative affect in these games. However, in multiline games, players receive attention-capturing feedback far more frequently (due to LDWs)—a factor which may prevent depressed minds from mind-wandering to negative places and ultimately induce flow and its accompanying positive affect. This may help explain why players prefer multiline games, and why there is greater flow experienced for multiline games than single-line games.

In addition to the relatively robust correlations between depression, mindfulness problems, and problem gambling status, there is also a relation between boredom and problem gambling. Boredom can be thought of as a subjectively unpleasant, negative state resulting from monotonous or dull situations (Merrifield & Danckert, 2014). However, researchers have also construed boredom in terms of attention—i.e., boredom occurs as a failure to engage attention with one’s environment (Eastwood et al., 2012). Problem gamblers score higher on self-report measures of boredom proneness than their non-problem counterparts. (Blaszczynski et al., 1990). Researchers have also shown that having a low tolerance for boredom is a significant factor in repetitive gambling behaviour (Blaszczynski et al., 1990). Researchers also report that being more susceptible to boredom and problem gambling may be in part a maladaptive coping strategy to deal with boredom and to escape dysphoric mood (Blaszczynski et al., 1986, 1990; Turner et al., 2006).

In the current experiment, we assessed a wide range of gamblers. Our goal was to examine if the more frequent reinforcing sights and sounds of the multiline slot machine serve to rein in the attention of minds that are prone to mind-wandering, fostering entry into “the zone,” and facilitate positive affect. We also sought to show that players would have a greater propensity to mind-wander during single-line play due to the prolonged losing streaks—a situation that should increase negative affect relative to the multiline game. Thus, we sought to test the following hypotheses: (1) we expected to see more flow, more positive affect, and less negative affect during multiline play than single-line play; (2) more instances of mind-wandering during single-line play than multiline play; (3) greater preference for multiline over single-line play; (4) based on previous research from our lab, we also expected to see a correlation between mindfulness problems in everyday life and problem gambling severity; (5) this correlation should be eliminated when we assess mindfulness during multiline slots play; and (6) show that depression, mindfulness problems, and boredom proneness are all correlated with problem gambling severity, but that dark flow can account for unique variance when predicting problem gambling status—over and above depression, mindfulness, and boredom proneness.

## Materials and Methods

### Participants

A total of 120 slot machine players were recruited from Elements Casino in Brantford, Ontario, Canada. The casino is a very popular 30,000 square foot venue with 539 slot machines and 48 table games. Recruitment was conducted from October 21, 2019 to November 1, 2019. Participants were pre-screened during recruitment to ensure that they were at least 19 years of age (the legal age to play a slot machine in Ontario), were not in treatment for problem gambling, and played a slot machine at least monthly. Ten participants were excluded for various reasons (e.g., falling asleep at the slot machine, for being intoxicated, withdrawing from the study early, etc.). This left 110 participants for analysis (56 female, 53 male, 1 non-binary). One participant did not disclose their age. The ages of the other 109 participants ranged from 22 to 82, with a mean of 59.93 years (*SD* = 13.46).

### Apparatus

#### Slot Machine Simulator

Participants played a five-reel, slot machine simulator housed in a slot machine casing so that it looked and played like an actual slot machine. Participants played two slot machine games, a multiline game and a single-line game. In the multiline game, participants played 20-lines on each spin and bet 1-cent per line (20 cents per spin). The 20-line playing session consisted of 300 spins, comprised of 197 losses, 40 wins, and 63 LDWs. In the single-line game, participants also bet 20 cents per spin (credits were worth 5 cents each and players bet 4 credits on the one line that they played). The playing session also consisted of 300 spins comprised of 284 losses and 16 wins. These relative frequencies of the different outcomes for both the single and multiline games were based on the programming documents of a commercially available machine, and the payback percentage (92.01%) was one commonly used in slot machines in Ontario. Outcomes in which participants lost their entire spin wager were followed by a lack of feedback (i.e., no sounds or animations) and winning outcomes were accompanied by auditory feedback and animations. The length of the sound was proportional to the win size.

### Materials

#### Demographic Questions

Participants completed demographic items regarding their age and gender.

#### Depression

Participants completed the depression subscale from the 21-item Depression, Anxiety, and Stress Scale (DASS-21; Lovibond & Lovibond, 1995). The depression subscale includes items such as, “I couldn’t seem to experience any positive feeling at all” and “I felt that I had nothing to look forward to.” Items were answered on a 4-point scale with the following options: (0) Did not apply to me at all, (1) Applied to me to some degree, or some of the time, (2) Applied to me to a considerable degree, or a good part of the time, and (3) Applied to me very much or most of the time. The seven-items were summed and multiplied by two to generate severity scores comparable to those of the DASS-42 (see Lovibond & Lovibond, 1995). The depression subscale of the DASS-21 has demonstrated excellent reliability (measured using Cronbach’s alpha, 0.88; Henry & Crawford, 2005).

#### Boredom Proneness Scale—Short Form (BPS)

The BPS (Struk et al., 2017) is an 8-item measure of trait boredom. The BPS contains items such as “Many things I have to do are repetitive and monotonous” and “Much of the time, I just sit around doing nothing.” Items were answered on a 7-point scale with the following options: (1) Strongly disagree, (2) Disagree, (3) Somewhat disagree, (4) Neither disagree nor agree, (5) Somewhat agree, (6) Agree, and (7) Strongly agree. The BPS demonstrates good internal consistency and comparable construct validity to the original Boredom Proneness Scale (Struk et al., 2017).

#### Canadian Problem Gambling Index (CPGI)

Participants also completed an item from the CPGI (Ferris & Wynne, 2001) that assesses the frequency in which players engage with slot machine gambling. The item was, “In the past 12 months, how often did you bet or spend money on slot machines or what some people call video lottery terminals (VLT)? When answering please base your answer on playing any kind of slot machine (i.e., a slot machine or VLT at either a physical or online casino.” They answered this item by choosing one of the following frequencies: daily, 2–6 times a week, about once a week, 2–3 times a month, about once a month, between 6 and 11 times a year, between 1 and 5 times a year, never, or I prefer not to say.

#### Problem Gambling Severity Index (PGSI)

The PGSI (Ferris & Wynne, 2001) is a nine-item screening tool that assesses gambling problems in the general population (Cronbach’s alpha of 0.84; Ferris & Wynne, 2001). Items were answered on a 4-point scale with the following options: (0) Never, (1) Sometimes, (2) Most of the time, and (3) Almost always. The nine-items were summed to produce a score for problem gambling (ranging from 0 to 27) with higher scores indicating greater risk for problem gambling.

#### Mindful Attention Awareness Scale (MAAS)

The MAAS (Brown & Ryan, 2003) is a 15-item questionnaire that assesses mindfulness in everyday life outside of gambling. The MAAS contains items such as, “I could be experiencing some emotion and not be conscious of it until sometime later” and “I tend to walk quickly to get where I am going without paying attention to what I experience along the way.” Items were answered on a 6-point scale with the following options: (1) Almost always, (2) Very frequently, (3) Somewhat frequently, (4) Somewhat infrequently, (5) Very infrequently, and (6) Almost never. The 15-items were averaged to produce a score for mindfulness with higher scores reflecting higher levels of dispositional mindfulness.

#### Game Experience Questionnaire (GEQ)

Participants completed three subscales (flow, positive affect, and negative affect) from the core version of the GEQ (IJsselisteign et al., 2013) to assess their experience of the slot machine and tone timing sessions. For (dark) flow, the items were: “I was fully occupied with the game,” “I forgot everything around me,” “I lost track of time,” “I was deeply concentrated in the game,” and “I lost connection with the outside world.” For positive affect, the following items were administered: “I felt content,” “I thought it was fun,” “I felt happy,” “I felt good,” and “I enjoyed it.” For negative affect, the follow items were administered: “It gave me a bad mood,” “I thought about other things,” “I found it tiresome,” and “I felt bored.” Items were answered using a 5-point scale with the following options: (0) Not at all, (1) Slightly, (2) Moderately, (3) Fairly, (4) Extremely. The items from each subscale were averaged to compute scores for positive affect and (dark) flow. The full GEQ has demonstrated good reliability, with a Cronbach’s alpha ranging from 0.71 to 0.89 for the various subscales included (IJsselsteijn et al., 2007).

#### Thought Probes

During both the single-line and multi-line slot machine sessions, participants were prompted with a thought probe after every 50 spins. The thought probe asked the participant to verbally indicate to the experimenter whether their thoughts were: on-game (i.e., thinking about the game), spontaneously mind-wandering (i.e., despite their best intentions to focus on the game, their mind had wandered), or deliberately mind-wandering (i.e., they intentionally chose to think about something else); see Seli, Risko, and Smilek (2016a, 2016b) and Seli, Risko, Smilek, and Schacter (2016a, 2016b) for further distinction between spontaneous and deliberate mind-wandering. The experimenter recorded the participant’s response on the tablet used for administering the survey measures. The total number of “on-game” responses were summed to produce an in-game mindfulness score with the scores ranging from 0 to 6 for each slot machine session.

#### Slot Machine Preference

At the end of play participants were asked, “What slot machine did you prefer playing?” They were asked to endorse one of the following options: the multiline slot machine, single-line slot machine, or neither.

### Design

The experiment employed a within-subjects design with all participants playing both games. Half of the participants played the multiline slot machine game first, followed by the single-line, whereas the other half of the participants played the single-line slot machine first. After the 600th spin a pop-up message appeared on the slot machine telling participants their final balance.

### Procedure

All participants approached the experiment station situated in the front lobby of the casino. After determining eligibility, participants were given an information synopsis of the study and gave written, informed consent before participating in this study. The University of Waterloo’s Office of Research Ethics approved all procedures in the study. Players were informed that they would be given a $25 Walmart gift card for participating, and that they would be able to win up to an additional $10.00 CAD (in cash) depending on their slot machine balance at the end of play. The simulator was pre-loaded with $20.00, and since all participants received the same outcomes, all participants ended up with $15.00 after the first game and $9.80 after the second game, regardless of their counterbalance order. The $9.80 was rounded up to $10.00 for each participant.

Using the online survey software Qualtrics, participants first completed the demographic questions, CPGI, PGSI, MAAS, depression questions, and BPS on a Lenovo tablet (model #TB-X103F). Participants then played either the multiline or single-line slot game (depending on their counterbalanced order). After completing each session, participants answered the positive affect, negative affect, and flow items of the GEQ on the tablet as well as the preference question. Participants were given a $25.00 Walmart gift card and their (rounded up) slot machine balance ($10.00 CAD for all participants). Participants were also given responsible gambling resources and the opportunity to take a feedback letter debriefing them of the studies purposes.

## Results

### Problem Gambling and Depression Scores

Using the interpretive categories of the PGSI suggested by Currie et al. (2013), the sample consisted of: 30 non-problem gamblers (PGSI score of 0), 52 low level problem gamblers (PGSI score ranging from 1 to 4), 15 moderate level problem gamblers (PGSI score ranging from 5 to 7) and 13 problem gamblers (PGSI score of 8 or greater). Using the interpretive categories of the DASS-21 suggested by Lovibond and Lovibond (1995), the sample contained a majority (*n* = 82) within the normal range of depression (scores of 0 to 9), 7 participants were characterized with mild depression (scores of 10 to 13), 11 with moderate depression (scores of 14 to 20), 5 with severe depression (scores of 21 to 27), and 4 with extremely severe depression (scores of 28 or more). One participant failed to fill out the entire DASS-21 and was not included in subsequent analyses involving depression. When filling out the slots frequency-of-play question, two participants indicated that they played less than once per month (despite our attempt to recruit players who played at least once per month or more), and one participant did not want to answer how often they played a slot machine.

### Game Preference

The majority of players (*n* = 83, 75.5%) preferred the multiline game, 21 players (19%) preferred the single-line game, and 6 players (5.5%) preferred neither game, χ^2^(2, *N* = 110) = 90.89, *p* < .001.

### Order Effects

In studies involving mind-wandering measures such as thought probes there might be a time-on-task effect. Therefore, it is crucial to assess whether there were effects of which game was played first. Since such effects could also influence affect and flow, for all planned analyses we first assessed whether there were order effects (a change in effect sizes depending on which game was played first). Where effects of order, or any interactions involving order were found, the files were split to directly compare those who played the multiline game first to those who played the single-line game first since this is the only comparison uncontaminated by order. A significant main effect of order was found for our in-game measure of mindfulness, *F*(1, 108) = 4.26, *p* = .042. We also found a significant main effect of order for our retrospective measure of negative affect, *F*(1, 108) = 4.52, *p* = .036, as well as an order by game interaction, *F*(1, 108) = 7.79, *p* = .006. There were no indications of order effects for the other measures (i.e., smallest *p* ≥ .054).

### Dark Flow

When comparing retrospective accounts of dark flow during multiline and single-line slots play, we included PGSI as a covariate in a repeated-measures analysis of covariance (see Delaney & Maxwell, 1981) because previous research has shown that problem gambling status positively relates to flow during slots play (Dixon et al., 2017, 2014, 2019a, 2019b; Murch et al., 2017). We found significantly greater flow during the multiline game (*M* = 1.42; *SD* = 1.02) than the single-line game (*M* = 1.32; *SD* = 0.99), *F*(1, 108) = 4.08, *p* = .046.

Retrospective dark flow ratings following multiline slots play were significantly correlated with: PGSI status *r*(108) = .195, *p* = .041, retrospective positive affect ratings *r*(108) = .623, *p* < .001, retrospective negative affect, *r*(108) = − .270, *p* = .004, and our in game measure of mindfulness during multiline slots play, *r*(108) = .346, *p* < .001. Similarly, retrospective dark flow ratings following single-line slots play was significantly correlated with PGSI status, *r*(108) = .245, *p* = .01, retrospective positive affect ratings during single-line play, *r*(108) = .586, *p* < .001, retrospective negative affect during single-line play, *r*(108) = − .341, *p* < .001, and our in game measure of mindfulness during single-line play, *r*(108) = .406, *p* < .001. We compared the magnitude of the correlations between retrospective dark flow ratings following multiline slots and PGSI status and retrospective dark flow ratings following single-line slots and PGSI status using Steiger’s *Z* (Steiger, 1980) and found that these two correlations were not significantly different, *Z*(107) = − 1.02, *p* = .31. A correlation matrix for all study variables is included in Table 1.

**Table 1:** Zero order correlations for all study variables

Variable	1	2	3	4	5	6	7	8	9	10	11
1. PGSI	–										
2. MAAS	− .478^***^	–									
3. Dep	.406^***^	− .578^***^	–								
4. BP	.293^**^	− .552^***^	.502^***^	–							
5. ML Mind	− .170	.060	− .084	.029	–						
6. SL Mind	− .213^*^	.092	− .190^*^	.006	.749^***^	–					
7. ML Flow	.195^*^	− .168	− .023	.054	.346^***^	.349^***^	–				
8. SL Flow	.245^*^	− .123	.006	.133	.310^**^	.406^***^	.862^***^	–			
9. ML Pos	− .039	.063	− .119	.022	.178	.235^*^	.623^***^	.535^***^	–		
10. SL Pos	.031	.041	− .081	.020	.112	.254^**^	.566^***^	.586^***^	.849^***^	–	
11. ML Neg	.080	− .100	.060	− .034	− .324^**^	− .343^***^	− .270^**^	− .230^*^	− .500^***^	− .445^***^	–
12. SL Neg	.125	− .066	.056	− .079	− .275^**^	− .424^***^	− .319^**^	− .341^***^	− .508^***^	− .514^***^	.751^***^

PGSI = Problem Gambling Severity Index; MAAS = Mindful Attention Awareness Scale; Dep = Endorsement of depression items on the DASS-21; BP = Boredom Proneness Scale; ML = Multiline slot machine; SL = Single-line slot machine; Mind = Number of “on game” responses; Pos = Endorsement of positive affect items on the GEQ; Neg = Endorsement of negative affect items on the GEQ

^*^p < .05; ^**^p < .01; ^***^p < .001

### Affect During Slots Play

Contrary to our prediction, we found no statistical difference in positive affect between the multiline slots game (*M* = 1.89; *SD* = 1.02) and single-line slots game (*M* = 1.87; *SD* = 0.99), *t*(109) < 1, *p* = .76. For negative affect, because there was an effect of which game was played first, we directly compared only those who played the multiline slot machine first to those who played the single-line slot machine first (the only contrast uncontaminated by order). We found that negative affect was significantly higher during the single-line game (*M* = 1.16; *SD* = 0.90) relative to the multiline game (*M* = 0.80; *SD* = 0.62), *t*(108) = 2.42, *p* = .017.

### Mindfulness

Since the number of on-task responses were found to be influenced by whichever game was played first, we once again restricted our analyses to compare those who played the multiline slot machine first to those who played the single-line slot machine first. We found that the number of “on-game” reports during the multiline game (*M* = 4.34; *SD* = 1.59) was significantly higher than during the single-line game (*M* = 3.32; *SD* = 2.32), *t*(108) = 2.68, *p* = .009. There were significantly fewer instances of spontaneous mind-wandering during the multiline game (*M* = 1.15; *SD* = 1.53) than the single-line game (*M* = 1.45; *SD* = 1.72), *t*(109) = 2.62, *p* = .010, but no significant difference in deliberate mind-wandering between the two games, *t*(109) < 1, *p* = .55.

Based on previous research, we expected to see a significant correlation between mindfulness (from the MAAS) and PGSI scores, however, when assessing mindfulness during slots play and PGSI scores, we expected to see a non-significant correlation (i.e., the correlation should disappear). In this study, we replicated the correlation between mindfulness from the MAAS and PGSI, *r*(108) = − .478, *p* < .001. When we assessed mindfulness during multiline slots play, we found that the correlation between mindfulness during slots and PGSI disappeared, *r*(108) = − .170, *p* = .077. Using Steiger’s *Z* (Steiger, 1980) we also showed that the correlation between mindfulness from the MAAS and PGSI was significantly different from the correlation between our in-game measure of mindfulness and PGSI, *Z* = − 2.56, *p* = .010. Although the correlation between mindfulness during single-line slots play and PGSI remained significant, *r*(108) = − .213, *p* = .025, it was also significantly different from the correlation between mindfulness measured by the MAAS and PGSI, *Z* = − 2.26, *p* = .024. Thus, it appeared as if our slot machine, and in particular our multiline slot machine, did indeed rein in the wandering mind.

### Hierarchical Regression Predicting PGSI

We used hierarchical regression in order to investigate whether the relatively newer concept of dark flow could account for unique variance when predicting problem gambling severity (over and above depression, mindfulness, and boredom proneness). Recall that the correlations between single-line and multiline dark flow and problem gambling status were not significantly different. To get the most stable estimate of dark flow during gambling, we combined participants dark flow ratings by averaging their retrospective multiline and single-line dark flow scores to create a total dark flow score. This score was used to predict PGSI scores in the last step of the hierarchical multiple regression after the more well-established measures had already been entered in prior steps. Specifically, depression ratings were entered at Step 1, mindfulness scores (from the MAAS) at Step 2, boredom proneness at Step 3, and total dark flow ratings at the final step. At Step 1, depression significantly contributed to the regression model, *F*(1, 107) = 21.10, *p* < .001, and accounted for 16.5% of the variation in PGSI score variance. At Step 2, mindfulness scores explained an additional 8.6% of the variation in PGSI scores and this increase in *R*^2^ was significant, Δ*F*(1, 106) = 12.11, *p* = .001. At Step 3, boredom proneness did not account for any additional variance in PGSI scores, Δ*R*^2^ = 0.0%, Δ*F*(1, 105) < 1, *p* = .90. At the final step, total dark flow ratings explained an additional 3.2% of the variation in PGSI scores—a significant change in the *R*^2^, Δ*F*(1, 104) = 4.69, *p* = .033. The overall regression model was significant when all four independent variables were included in Step 4, *F*(4, 104) = 10.25, *p* < .001, and accounted for 28.3% of PGSI status variance. For a full regression summary see Table 2.

**Table 2:** Hierarchical regression for variables predicting problem gambling severity

Model	*b*	*SE*	β	*R*^2^	Δ*R*^2^
*Step 1*				.165^***^	
Constant	1.88	0.43			
Depression	0.18	0.04	0.41^***^		
*Step 2*				.250^***^	.086^***^
Constant	9.28	2.86			
Depression	0.09	0.05	.20		
Mindfulness	− 1.44	0.41	− 0.36^***^		
*Step 3*				.250^***^	.000
Constant	9.51	2.86			
Depression	0.09	0.05	0.20		
Mindfulness	− 1.46	0.44	− 0.37^**^		
Boredom	− 0.01	0.05	− 0.01		
*Step 4*				.283^***^	.032^*^
Constant	7.85	2.91			
Depression	0.10	0.05	0.23^*^		
Mindfulness	− 1.30	0.44	− 0.32^**^		
Boredom	− 0.01	0.05	− 0.03		
Total Flow	0.70	0.32	0.18^*^		

Depression = Endorsement of the depression items of the DASS-21; Mindfulness = scores from the MAAS; Boredom = scores from the boredom proneness scale; Total Flow = Endorsement of the flow items from the GEQ, averaged between the multiline game and single-line game

^*^p < .05; ^**^p < .01; ^***^p < .001

## Discussion

Slot machines can create exceptional problems for some players. During slot machine play, some players describe a flow-like state which they call the “slot machine zone”— a complete immersion into slot machine play to the exclusion of all else. During this state of deep, effortless concentration, players report distortions of time and often describe this state as very pleasant.

In this study, we showed that, as predicted, players experienced greater flow during multiline play than during single-line play. For this analysis, we capitalized on previous research that shows that problem gambling severity significantly correlates with flow during play (Dixon et al., 2017, 2014, 2019a, 2019b, 2014; Murch et al., 2017). Therefore, we used (dark) flow as a covariate in order to reduce error variance. Although we found that players reported greater flow during the multiline game than the single-line game the effects were not as strong as anticipated (based on previous findings in our lab), and indeed we did not find stronger correlations between multiline dark flow and PGSI scores compared to single-line dark flow and PGSI scores—a pattern that has been shown previously (Dixon et al., 2017).

Even though players reported greater dark flow in the multiline game, contrary to our predictions, we found no statistical difference in positive affect when comparing between the multiline and single-line games. We also replicated a strong correlation between dark flow and positive affect while playing the multiline games. We did, however, find a difference in negative affect—players reported significantly more negative affect during the single-line game than the multiline game. One potential explanation for the failure to find differences in positive affect between the single-line and multiline games involves a trade-off between win size and win frequency across the games. In actual slot machine games (upon which our simulator was based), there were relatively frequent wins (*n* = 40) among the 300 spins of the multiline line game. By contrast, in the single-line game there were only 16 wins among the 300 spins. Despite this disparity in frequency—the overall amount of money paid back to the player was the same in both games. In slot machine parlance, the payback percentage of the two games did not differ (a situation which mimics real slot machines whose payback percentage does not change depending on how many lines are played). This matching of the payback percentage meant that when the infrequent wins in the single-line game did occur, their average size was far larger than the average size of wins encountered in the multiline game. The intermittent large wins in the single-line game may have caused dramatic fluctuations in positive affect. In other words, there may have been infrequent, but large “spikes” in positive affect caused by the large wins in the single-line game. It is well known that physiological arousal accompanies large wins in slot machine play (e.g., Dixon et al., 2014a, 2014b) presumably due to their inherently exciting properties. When polling participants after play, they may have taken into account both the long lulls in positive affect during losing streaks, but also the spikes in positive affect due to the excitement of the 16 relatively large wins. Thus, positive affect in the single-line game may have averaged out to the same value as the positive affect in the multiline game (which in part, may have been maintained by the smaller but far more frequent wins and LDWs).

While there was no difference in positive affect between games, the single-line game caused players to experience significantly greater negative affect. In terms of negative affect, the long chains of losses in the single-line game may have contributed to a lowering of mood relative to the multiline game where the rate of reinforcing feedback was far higher. In interpreting the more pronounced negative affect in the single-line games it is imperative to also consider mind-wandering. Recall that players reported more instances of mind-wandering during the single-line game (compared to the multiline game). If a wandering mind is an unhappy mind as suggested by Killingsworth and Gilbert (2010), it makes sense that one would see more negative affect in the single-line games—the long losing streaks provide more opportunities to mind-wander, which resulted in an increase in negative affect. This increase in negative affect may also explain why the majority of gamblers preferred the multiline game.

Another important finding concerning the greater degree of mind-wandering during the single-line game involves the *type* of mind-wander that players displayed. Players reported significantly more spontaneous mind-wandering (i.e., unintentional mind-wandering) during the single-line game than the multiline game but no significant difference in deliberate mind-wandering (i.e., intentional mind-wandering) between the two games. Thus, during the long losing streaks of single-line play, players found that their minds *unintentionally* wandered—perhaps to dark places.

Consistent with previous research, we found a negative correlation between mindfulness in everyday life and depressive symptomology outside of the gambling context. Such findings replicate previous studies (de Lisle et al., 2011; Dixon et al., 2017, 2014a, 2014b; Lakey et al., 2007; Reid et al., 2014). We also showed a correlation between mindfulness problems in everyday life and problem gambling severity (Dixon et al., 2019a, 2019b). Crucially, this correlation between mind-wandering and problem gambling disappears when mind-wandering is assessed during multiline slots play—a finding that replicates Dixon et al. (2019a, 2019b). It appears that multiline slot machine play (with its more frequent presentation of attention capturing feedback) is capable of reining in the wandering mind. A novel finding is that single-line slots play, with its long losing streaks, appears less effective at curtailing mind-wandering in these same players. Here we showed that the propensity to mind-wandering among problem gamblers (as shown by the correlation between MAAS and PGSI scores) was still evident when mind-wandering was assessed during single-line play.

Parallel findings emerged when we consider mind-wandering amongst those who were depressed. When mind-wandering is assessed in everyday life there is a strong correlation with depression. However, when mind-wandering is assessed during slot machine play, this correlation disappears during the multiline game but remains significant during single-line play. These assessments of mindfulness in everyday life versus our in-game measures of mindfulness while playing slots help bolster our previous assertions about escape gambling (see Dixon et al., 2019a, 2019b). If some players are prone to having their mind-wander to negative places, the frequent (but unpredictable) reinforcement of multiline slot machines may help rein in the wandering mind and prevent minds from unintentionally wandering to negative thoughts. Our findings show that less relief may be provided by single-line games. For both depressed individuals and problem gamblers, the propensity to mind-wander (likely during the long losing streaks) remains.

Another novel finding in this study involves the hierarchical multiple regression predicting problem gambling severity. We first replicated the positive correlations between problem gambling and depression (Dixon et al., 2017, 2014a, 2014b) problem gambling and mind-wandering (Dixon et al., 2019a, 2019b) and problem gambling and boredom proneness (Blaszczynski et al., 1990). These are well established relationships in the problem gambling literature. We also replicated the more novel relationship between problem gambling and (dark) flow (Dixon et al., 2017, 2014a, 2014b). When we used multiple regression to predict problem gambling scores, we found that retrospective ratings of total flow while playing slots significantly accounted for unique problem gambling severity variance, after accounting for depression, mindfulness, and boredom proneness. This indicates that there is something particular about this flow state that may be particularly nefarious for problem gamblers. The final model indicated that depression, mindfulness, and flow were significant predictors of problem gambling severity. This multifaceted relationship between depression, mindfulness problems, dark flow (and ultimately positive affect) may help elucidate why slot machines are especially appealing to those who gamble to escape. One possibility is that while some depressed players may play slots to modulate their arousal levels (Mercer & Eastwood, 2010), others (those who experience high levels of flow during play) may find that it elevates their mood. Thus, in this study, the depth of flow may be capturing those who gamble to escape—namely those who rectify their depressed mood via the positive affect they feel during a (dark) flow state. Further, gambling to escape may not be exclusively limited to depressed individuals. It may have some implications for any individual living with a mental health condition (e.g., anxiety, post-traumatic stress disorder, etc.)—being in the zone may not only prevent people from thinking about these negative states that characterize their lives, but also lead to elevations of positive affect.

Our study has some limitations. For example, we failed to replicate previous studies showing a positive relationship between dark flow during multiline slots and depression outside of the gambling context. We also failed to replicate a significantly larger correlation between dark flow and PGSI status for the multiline slots versus the single-line slots. Both limitations may be attributable to interrupting players during slots play to assess their “in-game” mindfulness. We likely broke the players flow state every time we interrupted them. This breaking of flow would have more profound effects in the multiline game. If depressed gamblers seek out and experience periods of unbroken flow when they normally play their favourite multiline slots game, when they play our multiline game (with its interruptions every 50 spins) it may have reduced the amount of flow they usually experience. Even though our data supported our prediction that players would experience greater flow during the multiline game than single-line game, the effect sizes were much smaller than anticipated—a limitation that is also likely attributable to the thought-probe methodology.

In conclusion, compared to single-line slots, it seems that multiline slot machines are capable of reining in the wandering mind by providing a highly captivating experience for the player. Depressed individuals may ruminate about their problems and seek relief from the frequent yet unpredictable reinforcement of the multiline slot machine. When compared to the less frequent feedback from single-line slots, the more frequent reinforcement provided by the multiline slot machine may foster greater entry into the “slot machine zone.” This complex relationship between problem gambling, mindfulness problems in everyday life, depression, and dark flow afforded by multiline slots may further help explain the motivations of those who gamble to escape.

## Appendix

See Tables 1, 2.
